# Actionable diagnostic delay in primary malignant bone tumors: a trigger-to-action framework for first-contact clinicians

**DOI:** 10.3389/fonc.2026.1963579

**Published:** 2026-09-22

**Authors:** Shu-Ze Tang, Wen-Jie Hong, Yin-Sheng Liang, Liang Ou

**Affiliations:** 1 Hunan University of Chinese Medicine, Changsha, China; 2 Affiliated Hospital of Hunan Academy of Chinese Medicine, Changsha, China

**Keywords:** actionable diagnostic delay, biopsy safety, bone sarcoma, first-contact clinicians, primary malignant bone tumor, referral threshold, treatment planning

## Abstract

Primary malignant bone tumors may initially resemble common musculoskeletal conditions, creating uncertainty about when first-contact clinicians should escalate care. This narrative review integrates selected guidelines, diagnostic-pathway studies, and imaging and biopsy literature to translate established principles into practical decisions before specialist referral. We propose actionable diagnostic delay as a descriptive, unvalidated framework: the potentially avoidable interval between a documented clinical trigger for escalation and initiation of the corresponding action. It complements, rather than replaces, established patient, diagnostic, referral, and treatment intervals. Concerning symptom trajectories, examination findings, aggressive or indeterminate imaging, and clinical-imaging-pathology discordance guide reassessment, imaging, referral, or coordinated biopsy. Plain radiographs are the first imaging step; persistent clinical suspicion despite normal radiographs warrants MRI or specialist referral. Referral should not await MRI when access is limited. A 2–4-week reassessment window is proposed only for mild, stable, low-suspicion presentations with adequate radiographs showing no suspicious or indeterminate lesion and is not an evidence-validated waiting period. Suspected fracture or acute neurological compromise requires emergency assessment. Metastatic carcinoma and myeloma are considered in parallel in the appropriate clinical context, while solitary or atypical aggressive lesions require a sarcoma-safe diagnostic pathway. Contemporary biopsy evidence supports specialist planning and radiology-pathology correlation, but diagnostic accuracy varies with case mix and sampling. Associations between diagnostic intervals and survival remain inconsistent and vulnerable to confounding by tumor biology. The framework therefore prioritizes diagnostic and procedural safety rather than a presumed survival benefit. Its performance, referral burden, and applicability across healthcare systems require prospective evaluation.

## Introduction

Primary malignant bone tumors are uncommon, accounting for less than 1% of all cancers in most epidemiological series (1–3). Osteosarcoma, chondrosarcoma, and Ewing sarcoma form the main diagnostic groups encountered in orthopedic oncology. Although their age distribution and anatomical predilection differ, they share a practical problem: the first presentation usually occurs outside a sarcoma service, where trauma, infection, degenerative disease, and benign bone lesions are far more familiar than cancer (4, 5).

Pain is usually the first complaint, but it is not always dramatic at the first visit. It may be intermittent, activity-related, or linked by the patient and clinician to a minor injury. Swelling, limp, a palpable mass, or pathological fracture may emerge later, and many patients are assessed before the clinical picture is complete (6–8). Ewing sarcoma may resemble osteomyelitis; low-grade cartilage tumors may resemble enchondroma or degenerative disease; and pelvic or spinal lesions may be interpreted as back pain, sciatica, arthritis, or an osteoporotic fracture. These are plausible first diagnoses. They become unsafe when they are maintained despite persistent or progressive symptoms.

Diagnostic delay in bone sarcoma has therefore remained a consistent feature of the care pathway. Recent reviews show wide variation in reported intervals, reflecting differences in tumor biology, location, healthcare systems, and definitions of delay (9, 10). In one referral-center study, the mean total diagnostic delay was 163.3 days for osteosarcoma and 160.2 days for Ewing sarcoma, but 688.0 days for chondrosarcoma (11). Associations between the diagnostic interval and survival are inconsistent and susceptible to the waiting-time paradox: aggressive tumors may prompt faster diagnosis while still having poorer outcomes (9–11). This review therefore distinguishes uncertain interval-related survival effects from the clinical need to reassess concerning symptoms, obtain a reliable diagnosis, and avoid unplanned procedures before specialist evaluation.

## Conceptual framework: actionable diagnostic delay

Terminology describing the pathway to cancer diagnosis is heterogeneous. The Aarhus Statement recommends defining explicit time points and intervals rather than using imprecise labels such as “patient delay” or “doctor delay” (12). In the sarcoma literature, the total interval commonly extends from first symptom to histological diagnosis and can be divided into a patient interval (symptom onset to first healthcare presentation) and a diagnostic interval (first presentation to diagnosis), with primary-, secondary-, and tertiary-care components; the treatment interval begins at diagnosis and ends when treatment starts (9, 10, 12). Referral intervals describe transfer between services and should likewise specify their start and end points. Some studies combine diagnosis and treatment initiation in their endpoint, further limiting comparability (13).

In this review, actionable diagnostic delay is an author-proposed, pragmatic descriptive framework. It does not replace these established intervals and is not presented as a validated cutoff, outcome measure, or surrogate for survival; a missed escalation opportunity is not itself proof of a delay-attributable adverse outcome. Rather, it identifies a potentially avoidable portion of the healthcare pathway that begins when a clinical trigger has reasonably indicated the need to escalate care and ends when the corresponding action is initiated. Relevant triggers include persistent or progressive focal symptoms after an initial benign assessment, concerning examination findings, an aggressive or indeterminate radiograph, high clinical suspicion despite unrevealing radiographs, or discordance among clinical, imaging, and pathological findings. The corresponding actions are structured reassessment, imaging escalation, specialist referral, or multidisciplinary review and planned biopsy.

Existing sarcoma evidence has not established a universally valid number of days, visits, or weeks that separates appropriate observation from harmful delay (9, 10). Actionability is therefore defined primarily by failure to respond to a documented trigger rather than by a single fixed time threshold. Urgency should be risk-adapted: aggressive imaging features, pathological fracture, a palpable mass, or neurological compromise require immediate or urgent escalation, whereas initially low-suspicion symptoms require a clearly scheduled review with explicit criteria for earlier reassessment. This trigger-to-action framework is used throughout the review and is operationalized in the diagnostic algorithm.

International sarcoma guidelines agree on early radiography, appropriate cross-sectional imaging, specialist-planned biopsy, and referral before definitive intervention (4, 14–16). Implementation differs between health systems: fragmented investigations and pre-referral procedures may be important in established networks, whereas cost, distance, and limited diagnostic capacity can constrain access elsewhere (17–20). Selected regional cohorts and an all-cancer systematic review illustrate these barriers but do not establish a global estimate of delay or a universal effect on survival (21–25).

## From guideline principles to first-contact decisions

Existing guidelines already provide core recommendations on presentation, imaging, referral, biopsy, and treatment (4, 14–16). The gap addressed here is therefore not an absence of guidance. Rather, these recommendations are embedded within comprehensive disease-management documents, are partly specific to individual referral systems, and are often applied only after suspicion of a primary malignant bone tumor has already arisen. First-contact clinicians face an earlier implementation problem: deciding when an ordinary musculoskeletal explanation has become unsafe, when a normal or equivocal radiograph should not close the pathway, how to safety-net a low-suspicion presentation, and how to escalate when advanced imaging or a sarcoma service is not immediately accessible. Limited familiarity with sarcoma work-up guidance among primary care practitioners reinforces this implementation problem (26).

The distinct contribution of this review is a cross-guideline, trigger-to-action synthesis for these upstream decisions. It links symptom trajectory, repeated presentation, examination findings, radiographic uncertainty, and clinical-imaging-pathology discordance to one of four actions: structured reassessment, imaging escalation, referral before intervention, or multidisciplinary review. It also separates tumor-specific mimics from cross-cutting red flags, distinguishes time-critical triggers from findings suitable for scheduled reassessment, and proposes resource-aware contingencies without replacing specialist guidelines. Where recommendations are directly guideline-based, they are described as such; where evidence does not support a universal threshold, pragmatic author interpretation is explicitly identified.

## Methods

This narrative review addresses early recognition, diagnostic delay, misdiagnosis, biopsy planning, and referral of primary malignant bone tumors. A targeted PubMed search for diagnostic delay and misdiagnosis was performed on September 3, 2026, using a publication-date limit through May 1, 2026. The export contained 868 unique PubMed records. The core search expression was:

(“Osteosarcoma”[Mesh] OR “Sarcoma, Ewing”[Mesh] OR “Chondrosarcoma”[Mesh] OR osteosarcoma*[tiab] OR chondrosarcoma*[tiab] OR “Ewing sarcoma*”[tiab] OR “Ewing tumor*”[tiab] OR “Ewing tumour*”[tiab] OR “bone sarcoma*”[tiab] OR “primary malignant bone tumor*”[tiab] OR “primary malignant bone tumour*”[tiab]) AND (“Delayed Diagnosis”[Mesh] OR “Diagnostic Errors”[Mesh] OR misdiagnos*[tiab] OR “diagnostic delay*”[tiab] OR “delayed diagnosis”[tiab] OR “delay in diagnosis”[tiab] OR “diagnostic interval*”[tiab] OR “time to diagnosis”[tiab] OR “symptom interval*”[tiab] OR “symptom duration”[tiab] OR “patient delay*”[tiab] OR “doctor delay*”[tiab] OR “referral delay*”[tiab] OR “delayed referral”[tiab])

Titles were reviewed, with selected abstracts and full texts assessed for relevance to clinical presentation, diagnostic pathways, imaging uncertainty, biopsy safety, and referral. Selection was purposive and non-exhaustive. Molecular or treatment-only reports were not selected unless relevant to these topics. The PubMed retrieval was supplemented by relevant sources already cited in the original manuscript and targeted literature searches during revision. Guideline, imaging, biopsy, and regional sources were checked against PubMed records, publisher pages, or official guideline documents; these checks did not constitute an exhaustive supplementary database search. The review cites 60 publications, including explicitly identified mixed-tumor, all-cancer, and methodological context; this is not a count of independent studies selected exclusively from PubMed.

Evidence was synthesized narratively, without independent duplicate screening, meta-analysis, formal risk-of-bias assessment, or certainty grading. Guidelines inform core imaging, referral, and biopsy principles; observational cohorts describe associations and pathway failures; the trigger-to-action framework and low-risk reassessment window are author interpretations. Interval definitions were informed by the Aarhus Statement and the sarcoma literature (10, 12).

## Why primary malignant bone tumors are missed

Missed or delayed diagnosis is rarely the result of a single error. More often, it reflects the intersection between a rare disease and very common musculoskeletal complaints. Most clinicians who first assess a patient with bone pain will see many cases of trauma, overuse injury, infection, arthritis, and benign bone lesions before encountering a primary bone sarcoma. That low prior probability shapes the first diagnostic impression (5, 26). In children and adolescents, the problem has been described as finding a rare diagnosis within a very crowded field of ordinary pain complaints (27). A painful knee in an adolescent is easily framed as sports-related; hip or pelvic pain may be interpreted as referred spinal or joint disease; and a slowly progressive cartilaginous lesion may be followed as benign until pain, cortical change, or soft-tissue extension becomes difficult to ignore.

The clinical presentation does little to protect the patient from an ordinary diagnosis. Pain is the earliest and most consistent symptom in many patients, but early pain is not always continuous, nocturnal, or alarming. In a high-grade bone sarcoma cohort, pain was recorded in 81.4% of patients with osteosarcoma, 68.9% with Ewing sarcoma, and 68.4% with chondrosarcoma (11). George and Grimer found that 36 of 41 patients with bone sarcoma (88%) had features at first healthcare contact that should have prompted further investigation; only 4 patients (10%) were referred directly to a specialist sarcoma unit (28). These data point away from late presentation alone and toward a failure to escalate when symptoms persist or evolve.

A related problem is diagnostic anchoring. Once a benign explanation has been recorded, later visits may confirm rather than challenge it. Analgesics, physiotherapy, antibiotics, or immobilization may produce partial or temporary improvement, which can reinforce the initial impression. The opportunity for diagnosis is then not the first visit alone, but the second or third contact, when the symptom course should be reconsidered. A benign diagnosis should become less secure when pain becomes focal, nocturnal, progressive, or disproportionate to examination findings.

Tumor biology and anatomical site add another layer of ambiguity. Osteosarcoma commonly arises in the metaphyses of long bones, especially around the knee; an injury-related history in a young patient may favor an initial diagnosis of sports injury or tendinopathy (6, 14). Ewing sarcoma can resemble osteomyelitis, and routine laboratory findings may not reliably distinguish infection from tumor (29). Chondrosarcoma presents a different problem, described in clinical and imaging literature (30, 31). Its course may be indolent, and distinguishing enchondroma from an atypical cartilaginous tumor (ACT) or grade 1 chondrosarcoma can be difficult even for experienced radiologists and pathologists (30, 31). For low-grade central cartilaginous tumors, WHO terminology uses ACT in the appendicular skeleton and grade 1 chondrosarcoma at axial sites, including the pelvis, scapula, and skull. ACT is classified as intermediate and locally aggressive; it is discussed here as a diagnostic differential rather than a separate malignant subtype (14).

The site of disease often determines the story the clinician hears. Pelvic, sacral, spinal, and proximal femoral tumors may present as back pain, sciatica, hip disease, disc pathology, or osteoporotic fracture. These diagnoses are common and usually correct in general practice; for that reason, they are hard to abandon. In pelvic bone sarcoma, symptoms may be prolonged and non-specific, with inaccurate diagnoses including disc disease, bursitis, stress fracture, hernia, urinary infection, and degenerative arthritis (32). The aim is not to turn every episode of bone pain into a cancer work-up. It is to reassess focal, persistent, progressive, or unexplained pain before the original benign label becomes a barrier to diagnosis. **Table 1** summarizes characteristic mimics and interpretive pitfalls by tumor subtype or site; cross-cutting escalation triggers and actions are presented separately in **Table 2**.

**Table 1:** Characteristic mimics and interpretive pitfalls by tumor subtype or site.

Tumor type or site	Typical misleading diagnosis	Subtype- or site-specific pitfall
Osteosarcoma near the knee	Sports injury or tendinopathy	A metaphyseal lesion near a major joint in a young patient may be attributed to recent minor trauma or an overuse condition, reinforcing a benign label (6, 14).
Ewing sarcoma	Osteomyelitis	The infection-like presentation can anchor the diagnosis; routine laboratory findings alone may not reliably distinguish the two conditions (29).
Atypical cartilaginous tumor (ACT)/grade 1 chondrosarcoma	Enchondroma	Cartilage matrix and low-grade histology overlap with enchondroma. For central lesions in this low-grade spectrum, terminology is site-dependent: appendicular ACT is intermediate and locally aggressive, whereas axial lesions are termed grade 1 chondrosarcoma. An indolent course or chondroid appearance alone does not resolve the distinction (14, 30, 31).
Pelvic bone sarcoma	Lumbar disc disease, spinal stenosis, hip arthritis, or bursitis	Hip, buttock, groin, or radicular symptoms may divert assessment toward lumbar or joint disease, obscuring the pelvic origin (32).

Sources: subtype- and site-specific examples from clinical, imaging, and presentation literature (6, 14, 29–32). These are interpretive pitfalls, not diagnostic criteria; cross-cutting escalation triggers and actions appear in **Table 2**.

**Table 2:** Cross-cutting escalation triggers, contextual modifiers, and practical responses.

Domain	Escalation trigger or context	Practical response
Pain pattern	Persistent, progressive, night/rest, or recurrent focal bone pain.	Obtain two-plane radiographs. A normal result must not prevent urgent MRI or specialist referral when clinical suspicion persists (14, 34).
Physical findings	Palpable or enlarging mass, objective swelling, progressive loss of movement or limp, neurological deficit, or suspected pathological fracture.	Arrange urgent imaging and specialist assessment, not observation. Suspected fracture or acute neurological compromise requires emergency specialist coordination. For suspected tumor-related fracture, immobilize and provide analgesia; avoid unplanned internal fixation (14).
Imaging	Aggressive or indeterminate lesion; wide transition zone, destructive pattern, aggressive periosteal reaction, cortical breach, or soft-tissue extension; clinical–imaging discordance.	Obtain urgent MRI and refer before local biopsy, curettage, fixation, or excision. If MRI is unavailable or would materially delay care, refer without waiting (14, 34).
Clinical course	Initial observation is restricted to mild, stable, short-duration symptoms, adequate radiographs showing no suspicious or indeterminate lesion, and no high-suspicion features.	Schedule clinician-led reassessment within 2–4 weeks† and document escalation criteria. Failure to improve, progression, repeated presentation, or diagnostic discordance requires MRI or referral without another observation cycle.
Age and systemic context	Age >40 years, previous cancer, multiple lesions, or myeloma clues modify the differential; age alone is not a red flag.	Investigate metastases/myeloma in parallel without delaying indicated referral. Multiple typical lesions with a compatible known primary may follow a metastatic pathway; solitary, atypical, or aggressive lesions need sarcoma-team assessment before local biopsy or fixation (14).

† The 2–4-week interval is proposed by the authors for operational safety-netting in otherwise low-risk presentations with adequate radiographs showing no suspicious or indeterminate lesion; it is not a validated cutoff and must not delay MRI or referral when any high-suspicion feature is present. Red flags guide escalation, not estimation of the survival effect of a diagnostic interval.

Sources: adapted from bone sarcoma and imaging guidance (14, 34). The reassessment interval is author-proposed. Use local urgent or emergency pathways according to clinical risk; this table is not a validated prediction rule.

Jaw osteosarcoma illustrates a site-specific trap. A Dutch series of 20 patients described misleading dental presentations and repeated treatment or observation before recognition (33). Among cases classified as delayed, five biopsies had initially been interpreted as fibrous dysplasia, osteoma, or pleomorphic adenoma. Small or fragmented specimens and incomplete clinicoradiographic information were proposed contributors. Inconsistent reporting of the delay threshold and individual intervals prevents a reliable delay-rate estimate, and the study could not assess prognosis. Its contribution is therefore mechanistic, not a generalizable estimate of frequency or survival.

## Clinical and imaging red flags

The clinically important signal is the symptom trajectory, not a requirement to accumulate a fixed number of red flags. Focal pain that persists beyond the expected recovery course, progresses, recurs, or is accompanied by swelling or functional loss warrants reassessment (6–8). Night pain increases concern, but its absence does not exclude sarcoma: in the Swedish presentation study, night pain was reported in only 21% of osteosarcoma and 19% of Ewing sarcoma cases at the first visit (6). These referral and registry data describe presentation among affected patients, not the predictive value of symptoms in an unselected primary-care population.

Several clinical findings should shorten the interval to imaging and referral. In George and Grimer’s bone sarcoma cohort, deep bony pain was present in 88% at first presentation, worsening pain in 41%, visible swelling in 49%, night pain in 34%, and limp or difficulty walking in 29% (28). A palpable mass, reduced range of motion, neurological symptoms, or pathological fracture also warrants prompt assessment, with urgency determined by the clinical findings and risk (14). Fever and inflammatory markers may support infection, but Ewing sarcoma may present in the same way (29). Conversely, normal blood tests do not exclude malignancy (14). The more useful clinical question is whether symptoms are focal and evolving, and whether the physical findings match the proposed benign diagnosis.

Plain radiographs are the recommended initial imaging test for suspected primary bone tumors (14, 34). Interpretation should assess lesion location, destruction pattern, transition zone, periosteal reaction, cortical integrity, and matrix mineralization (5, 35). Permeative or moth-eaten destruction, aggressive periosteal reaction, cortical breakthrough, or a soft-tissue mass warrants evaluation for a potentially malignant lesion, although infection and aggressive benign lesions can overlap. Imaging concern should trigger expert characterization rather than a presumptive histological diagnosis.

Indeterminate findings require correlation with the clinical course and prior imaging, with musculoskeletal radiology review when these disagree. The Bone-RADS consensus provides management categories for incidental solitary lesions on CT or MRI in adults (36). It should not be presented as a validated rule for symptomatic children, a general primary-care screening tool, or a substitute for specialist judgment.

MRI is the key next step when radiographs are indeterminate or aggressive, or when symptoms remain concerning despite unrevealing radiographs (4, 14, 34). It defines marrow involvement, soft-tissue extension, skip lesions, joint proximity, and neurovascular relationships, and it informs biopsy and surgical planning (4, 14). CT is complementary for mineralized matrix, cortical destruction, complex anatomy, or pulmonary staging and can help local assessment when MRI is contraindicated (14, 34). Bone scan, PET/CT, or whole-body MRI may be used for staging according to tumor type and local protocols, but they should not substitute for adequate local MRI of the involved region (4, 14).

**Table 2** links cross-cutting triggers to the next action, while **Table 1** retains subtype- and site-specific pitfalls. The age/systemic-context row modifies the differential diagnosis; age alone is not an escalation trigger.

## Distinguishing primary bone sarcoma from metastatic and hematologic disease

Although this review focuses on primary malignant bone tumors, first-contact assessment of an aggressive bone lesion must include metastatic carcinoma and hematological malignancy. In adults, particularly those older than 40 years, bone metastases and plasma cell neoplasms are substantially more common than primary bone sarcoma (14, 37). A prior malignancy, multiple lesions, and a distribution typical of skeletal metastases increase the probability of secondary disease; however, age or a cancer history does not by itself establish that a solitary lesion is metastatic.

Initial evaluation should combine the lesion’s local behavior with the systemic context. Plain radiographs remain the first local study. An indeterminate or aggressive lesion on radiographs warrants MRI of the involved region, with CT used when appropriate for further characterization or procedural planning. Persistent pain or a palpable mass despite unrevealing radiographs should prompt urgent MRI or specialist referral (14, 34). In adults older than 40 years, prompt investigation for an occult primary or plasma cell disorder may include CT of the chest, abdomen, and pelvis, whole-skeleton imaging, and a myeloma screen, provided that these investigations do not delay referral (14).

The practical handover rule is based on diagnostic certainty rather than age alone. A patient with a known carcinoma and multiple lesions typical of skeletal metastases can usually enter an appropriate metastatic-cancer pathway. By contrast, a solitary aggressive lesion, an atypical lesion in a patient with cancer, or a lesion without an identified primary should be referred to a bone sarcoma or orthopedic oncology service before biopsy, curettage, or fixation. This preserves the biopsy tract and surgical options if the lesion proves to be a primary sarcoma and avoids an oncologically inappropriate unplanned procedure. This distinction follows the specialist referral principles in current bone-sarcoma guidance (14).

## Biopsy pitfalls, referral, and multidisciplinary evaluation

Biopsy is often described as the final diagnostic step, but in bone sarcoma it is also the first treatment-planning step. A biopsy establishes histology and grade, enables molecular or ancillary testing when required, and guides chemotherapy, radiotherapy, and surgery. It can also create harm: tissue planes may be violated, and a poorly placed incision or needle path may force a wider resection than would otherwise have been necessary (38, 39). Biopsy-tract contamination is documented, although reported rates vary with technique, case selection, and ascertainment (40, 41). The route should therefore be planned as part of definitive surgery. For this reason, biopsy should be planned by, or in direct consultation with, the team that will perform definitive treatment.

Several principles are consistent across guidelines and classic orthopedic oncology teaching. Necessary local cross-sectional imaging, usually MRI, should precede biopsy to guide sampling and the biopsy route. Systemic staging is coordinated by the specialist team according to the suspected or confirmed tumor type; completion of every staging investigation is not a universal prerequisite for biopsy (14). The biopsy route should be short, direct, and placed within the planned surgical field so that it can be removed en bloc with the tumor. It should avoid neurovascular bundles, uninvolved compartments, joints, and skin flaps required for reconstruction. Hemostasis matters because a hematoma may contaminate tissues beyond the biopsy path. Multiple samples should be obtained from viable tumor, avoiding necrotic or hemorrhagic areas when possible (14, 39). The main precautions before biopsy or surgery are summarized in **Table 3**.

**Table 3:** Do’s and don’ts before biopsy or surgery.

Step	Do	Do not	Rationale
Before biopsy	Refer early; review clinical findings and radiographs; obtain local cross-sectional imaging before biopsy; coordinate systemic staging separately with the sarcoma team	Do not ‘settle’ a suspicious lesion by unplanned local biopsy or surgery; do not routinely defer biopsy until all systemic staging is complete	Pre-biopsy imaging and specialist planning preserve staging accuracy and surgical options; contemporary core-needle biopsy evidence supports coordinated radiology-pathology input (14, 42, 43)
Biopsy planning	Plan the tract with the treating tumor team; keep it short, direct, and within the future resection field	Do not cross uninvolved compartments, joints, neurovascular bundles, or reconstructive flaps	The tract may be contaminated and should be removable with the tumor
After pathology	Correlate histology with imaging and clinical behavior in MDT; repeat biopsy if results are discordant	Do not accept a benign or non-diagnostic result when imaging remains aggressive	Sampling error and diagnostic discordance remain possible; results that conflict with imaging or clinical behavior require expert review (43, 44)

Sources: core planning principles from bone-sarcoma guidelines and biopsy reviews (4, 14, 39, 56); procedural and diagnostic context from historical and contemporary studies (38, 40–44, 46). This is a clinical synthesis, not a formally graded recommendation set.

The type of biopsy depends on site, imaging features, local expertise, and the diagnostic question. Image-guided core-needle biopsy is an effective first-line option when planned by an experienced musculoskeletal oncology team and coordinated with radiology and pathology (14, 39, 42, 43). Open biopsy remains appropriate in selected cases, but it should not be an exploratory procedure. Fine-needle aspiration alone is usually insufficient for primary bone tumors because architecture, immunohistochemistry, and molecular studies may be needed. Technique selection should balance tissue adequacy with preservation of future surgical options.

A 2023 meta-analysis of 29 studies (published in 1996-2021) reported pooled diagnostic yield of 91.4% and accuracy of 92.7% for core-needle biopsy across benign and malignant bone tumors (42). In a 2016–2020 referral-center cohort of 196 suspected primary bone tumors, diagnostic yield was 84.7%, accuracy among operated lesions was 91.7%, and the overall complication rate was 1.0% (two hematomas) (43). Diagnostic yield and accuracy are distinct outcomes, and these estimates depend on case mix and reference standards; they should not be treated as universal primary-sarcoma rates or directly compared with historical biopsy series.

Pathology should be read as part of the case, not as an isolated label. A benign or non-diagnostic biopsy result cannot safely close the pathway when imaging is aggressive or the clinical course is concerning. Sampling error, necrosis, reactive bone, prior fracture, infection, and low-grade cartilage lesions can all complicate interpretation. In such cases, review of imaging and histology together is essential, and repeat biopsy may be safer than proceeding on a result that does not explain the lesion. This concordance-based approach is supported by guidelines and contemporary biopsy/network studies (14, 43, 44).

Telangiectatic osteosarcoma provides a concrete example of sampling discordance. In a retrospective series of 26 surgically confirmed cases, preoperative core-needle biopsy was interpreted as an aneurysmal bone cyst in nine and was non-diagnostic in three; ten were identified as conventional osteosarcoma and four were correctly subtyped (45). Thus, failure to identify the exact subtype should be distinguished from failure to recognize malignancy. Samples dominated by blood-filled spaces could miss viable malignant cells in the peripheral or septal tissue. Among 22 patients with MRI, thick peripheral/septal enhancement and cortical destruction with a soft-tissue mass were reported in all cases, illustrating why a benign cystic interpretation must be reconciled with aggressive imaging. The series lacked a benign comparison group and does not establish imaging specificity or a general false-negative rate for core-needle biopsy.

Referral before biopsy is not a matter of institutional preference; it is part of oncologic safety. The Mankin series, published in 1996, remains a historical warning about poorly planned biopsy rather than an estimate of present-day risk; its error and amputation figures predate current image-guided practice and should not be extrapolated to contemporary pathways (38). Referral studies also show that patients may undergo advanced imaging, biopsy, or even surgery before reaching a sarcoma service, and that such pre-referral activity can delay diagnosis or complicate later management (17, 18, 46). A suspicious bone lesion should be referred before it is cut, scraped, fixed, or excised.

A historical Korean series further separates failures to obtain pathology from errors in its interpretation. Kim et al. described 26 patients with high-grade osteosarcoma selected for an inappropriate initial procedure and a diagnostic interval exceeding 45 days (47). Tissue had not been submitted for pathological examination in 12 cases, whereas an incorrect pathological diagnosis had been recorded in 14. At referral, 12 patients had distant metastases. However, all patients had experienced both an inappropriate procedure and delay, and there was no non-delayed control group; their individual contributions to the poor outcomes could not be separated. These selected cases support attention to planned tissue diagnosis and diagnostic concordance, but do not provide a causal estimate of the survival effect of delay or a population error rate.

Contemporary network data support specialist diagnostic coordination, although they measure pathology discrepancies rather than biopsy complications. In a French nationwide cohort of 1,075 primary bone tumors, initial pathological assessment within an expert sarcoma network was associated with fewer major diagnostic discrepancies (7% versus 57%) and a shorter mean final diagnostic delay (2.8 versus 6.5 weeks) than assessment outside the network (44). These observational findings do not establish a causal effect of referral, but reinforce the need for expert clinicoradiological-pathological correlation. Modern biopsy techniques do not eliminate sampling error or diagnostic discordance; a benign or non-diagnostic result that conflicts with the clinical course or imaging requires expert review and, when indicated, repeat biopsy (14, 43, 44).

Multidisciplinary review integrates tumor extent, histological classification, surgical options, and systemic or local treatment planning (14). Evidence for service organization must be distinguished by population. A French study of soft-tissue and visceral sarcomas associated pre-treatment specialist board review with better guideline adherence and relapse-free survival (48). A later nationwide mixed-sarcoma study associated surgery in reference centers with improved outcomes (49). These observational findings support specialist coordination but are not bone-sarcoma-specific causal estimates. The bone-tumor diagnostic-network evidence described above is more directly relevant to diagnostic concordance (44).

## Discussion: diagnostic intervals, the waiting-time paradox, and pathway safety

The relationship between diagnostic interval and outcome is not uniformly linear. The waiting-time paradox describes a potential inverse association in which biologically aggressive tumors cause earlier or more alarming symptoms, prompt faster presentation and investigation, and nevertheless have worse outcomes. More indolent tumors may have longer symptom histories but more favorable outcomes. Tumor biology and symptom severity can therefore influence both the interval and prognosis, confounding comparisons of shorter and longer intervals (10, 11). Such findings do not mean that waiting is protective, just as a null association does not prove that avoidable delay is harmless.

The clinical evidence illustrates this uncertainty. In prospectively collected multicenter data from 436 patients younger than 21 years with Ewing bone tumors, Brasme et al. found no association between time to diagnosis and metastasis, surgical outcome, or survival; associations with tumor volume and histological response seen in univariable analysis did not persist after adjustment (50). A retrospective referral-center study of 102 high-grade bone sarcomas likewise found no significant survival disadvantage for total intervals of at least four months (11). These cohort findings do not establish a safe observation period for an individual with concerning symptoms, and heterogeneous interval definitions and case mix limit generalization across bone-sarcoma subtypes (9, 10).

Further cohorts illustrate why interval definitions and adjustment matter. In an Indian retrospective cohort of 1,227 patients with osteosarcoma or Ewing sarcoma, more than four months from symptom onset to histopathological confirmation was not significantly associated with overall or event-free survival (51). In prospectively collected data from 220 patients with osteosarcoma around the knee in China, survival differed across symptom-interval groups in unadjusted analyses, but the association was not statistically significant in the multivariable model (P = 0.182) (52). Conversely, a UK retrospective cohort of 250 children with osteosarcoma found that more than four weeks from symptom onset to the first specialist assessment was associated with poorer overall survival after adjustment (hazard ratio, 2.10; 95% confidence interval, 1.18-3.77), whereas a comparison using an eight-week cutoff was not significant (53). Specialist assessment, histological confirmation, and treatment initiation are not interchangeable endpoints. Differences in case selection and covariate adjustment, including adjustment for variables that may lie on the causal pathway, further limit interpretation of these observational associations.

An adult Mexican cohort of 96 patients also reported poorer survival with more than five months from symptom onset to histopathological diagnosis (54). However, that threshold was selected from the same cohort using receiver operating characteristic analysis, the groups differed in tumor site and histological composition, and the text and outcome table reported inconsistent hazard-ratio estimates. The result therefore warrants cautious qualitative interpretation rather than adoption of its effect size or cutoff. Neither a study-specific threshold nor a statistically non-significant association establishes a safe period of observation.

Diagnostic-pathway quality should be distinguished from elapsed time alone. Local extent, neurovascular involvement, and pathological fracture can complicate limb-salvage planning (4, 14). A meta-analysis associated pathological fracture with poorer survival in localized high-grade osteosarcoma, but that association does not establish that delay caused the fracture or the survival difference (55). By contrast, an unplanned curettage, fixation, or excision may directly disrupt tissue planes and constrain subsequent oncologic surgery (38, 39, 46). Avoiding such procedures is a procedural-safety rationale for coordinated referral, not evidence that every additional diagnostic day worsens survival.

## Evidence across healthcare settings

Pathway barriers vary across settings. In a South African tertiary-hospital series of 61 patients with osteosarcoma, the mean interval before arrival at the tumor unit was 4.5 months; limited MRI access also delayed biopsy after arrival (21). A Peruvian two-center cohort of 113 children reported a median diagnostic latency of 13.5 weeks, without an association between diagnostic latency and survival; delays in treatment completion, often related to unavailable beds, were associated with poorer outcomes (22). A Malaysian series of 30 patients with osteosarcoma around the knee separated presentation-to-radiography, radiography-to-referral, and specialist work-up intervals (23). These reports identify distinct bottlenecks rather than comparable national delay estimates.

Contextual evidence has a broader but less specific scope. An Egyptian childhood-cancer cohort included only 12 bone tumors and cannot provide a bone-sarcoma-specific estimate (24). Selected Indian reports describe financial, travel, awareness, and referral barriers, while Indian guidance discusses resource-adapted management (19, 20, 56, 57). An all-cancer systematic review across 57 low- and middle-income countries found barriers across access, diagnosis, and treatment, with substantial methodological heterogeneity (25). This coverage illustrates several health systems, not global representativeness.

Separating pathway stages can identify different targets for improvement. A Shanghai series of 82 patients with osteosarcoma around the knee reported median intervals of 1.05 months from symptom onset to first medical consultation and 0.45 months thereafter; its methods defined diagnosis by the biopsy-report date (58). The series excluded one exceptionally long interval and assessed time to lung metastasis, not overall survival. Its recruitment site and period overlap with part of the later Chinese cohort (52), so the two reports should not be assumed to provide wholly independent patient evidence. In a Polish series of 32 primary bone tumors, including six giant cell tumors, the median interval from symptoms to suspicion and referral was seven months (59). Nineteen patients underwent radiography at the first visit, and nine of those 19 were then referred to an oncology center. These mixed-cohort findings illustrate separate imaging and referral steps, not a primary-sarcoma-specific referral rate. Likewise, time from histological diagnosis to surgery should not automatically be classified as avoidable delay when planned neoadjuvant treatment may occupy that interval (59).

Age-group comparisons also need to be separated from the effects of diagnostic timing. In a Mexican pediatric cohort of 149 patients with osteosarcoma, the median symptom-to-diagnosis interval was seven weeks in children younger than ten years and twelve weeks in adolescents, without a statistically significant difference in overall survival between age groups (60). This does not establish the independent effect of the diagnostic interval. Treatment abandonment was reported in 56 of 149 patients (37.6%), emphasizing that earlier recognition and referral alone cannot address all interruptions later in the care pathway.

A practical implementation task is to make the handover observable: document the working diagnosis, the next responsible service, the planned review date, and the findings that require earlier return. Where specialist or imaging access is constrained, transmitting existing radiographs and arranging remote advice may support escalation. Referral initiation, receipt, and completed assessment should be recorded separately; sending a request alone does not establish that the patient reached care. These are proposed workflow measures, not interventions with demonstrated effectiveness in this review.

## Practical diagnostic algorithm

**Figure 1** combines guideline-informed diagnostic actions with the author-proposed safety-net branch. Emergency assessment applies to suspected pathological fracture or acute neurological compromise; other suspicious clinical or imaging findings require urgent specialist assessment according to local pathways. MRI and referral should be coordinated in parallel, and lack of prompt MRI access must not prevent referral (14, 34). These urgency labels reflect clinical and procedural risk rather than a validated survival-related deadline.

**Figure 1:**
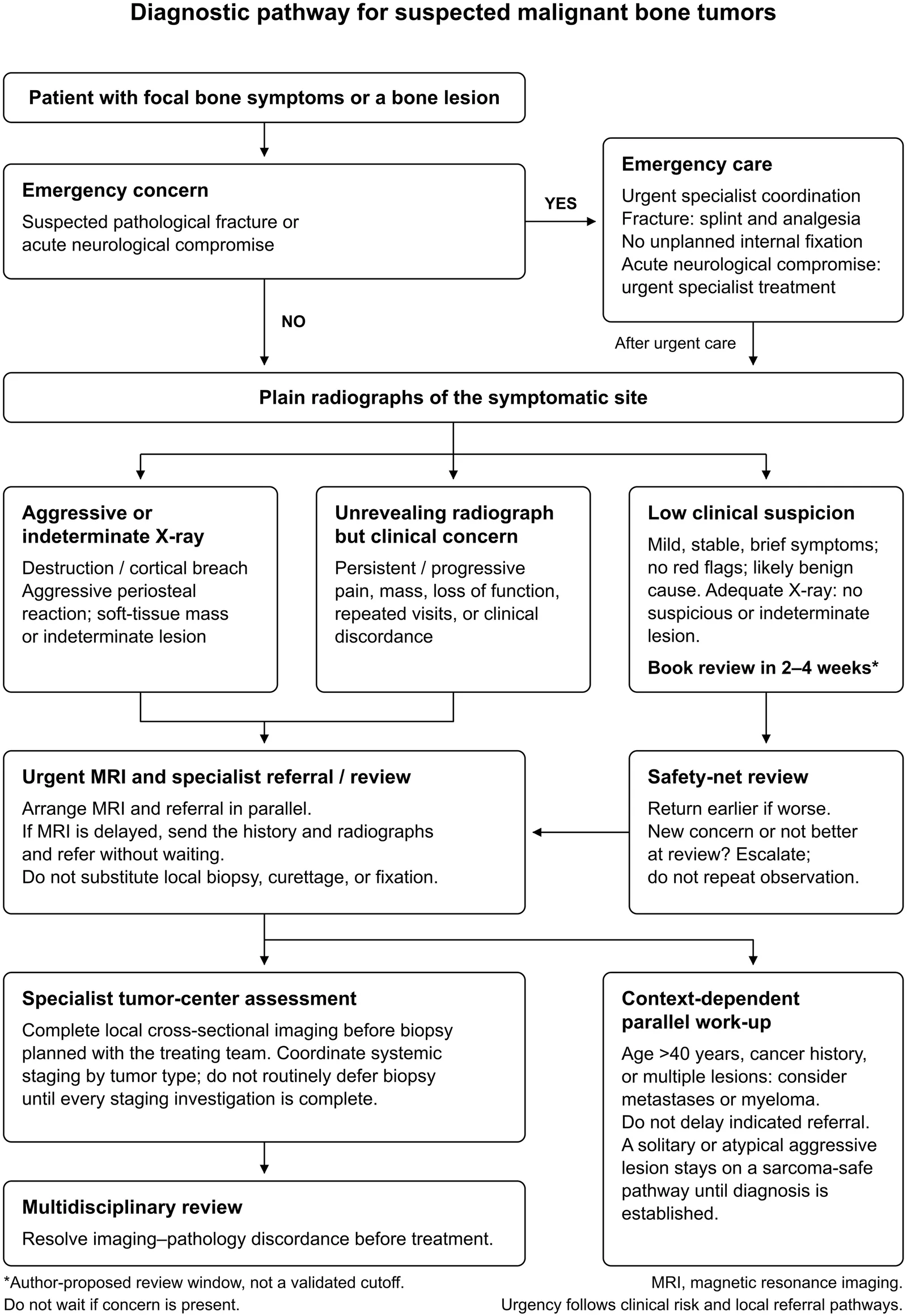
Guideline-informed diagnostic and referral pathway with author-proposed safety-netting. The 2–4-week window applies only to low-suspicion presentations with adequate radiographs showing no suspicious or indeterminate lesion and is not a validated cutoff. Urgency reflects clinical and procedural risk, not a proven survival deadline. Systemic investigations depend on context; they must not delay indicated referral. Emergency care takes precedence over routine diagnostic sequencing.

Systemic evaluation for metastases or myeloma depends on age, cancer history, lesion distribution, and specialist advice. As detailed above, a compatible metastatic presentation may follow a disease-specific pathway, but a solitary or atypical aggressive lesion remains on the sarcoma-safe pathway until its diagnosis is established (14).

Once malignancy is suspected, referral and specialist-directed systemic staging should be coordinated according to the suspected or confirmed tumor type, without delaying referral. Chest CT is commonly required for pulmonary staging in osteosarcoma and Ewing sarcoma; additional systemic imaging depends on tumor type and institutional protocol (4, 14, 16). Biopsy should follow the necessary local cross-sectional imaging and be planned with the treating team; it should not routinely be deferred until every systemic staging investigation is complete. After histology is available, the case should be reviewed in a multidisciplinary setting to confirm concordance between clinical presentation, imaging, and pathology, and to plan systemic therapy, surgery, radiotherapy, or surveillance as appropriate.

The referral should include symptom trajectory, previous images and reports, treatments, and prior procedures. Local biopsy or surgery while awaiting review should be avoided unless coordinated with the sarcoma team. For suspected tumor-related pathological fracture, provide temporary immobilization or external splintage and analgesia while urgently coordinating imaging and biopsy with the sarcoma team; avoid unplanned internal fixation. Necessary emergency treatment for acute neurological compromise must not be withheld and requires urgent specialist coordination (14).

For mild, stable, short-duration focal symptoms, adequate radiographs showing no suspicious or indeterminate lesion, and no high-suspicion feature, we propose a booked clinician-led review within 2–4 weeks with earlier return for deterioration. This is a pragmatic author-proposed window, not a validated threshold or a requirement to wait. Persistent or progressive symptoms, a mass, concerning examination findings, repeated presentation, or imaging discordance should instead trigger MRI or specialist referral without another observation cycle (14, 34, 37). The window should be shortened when the clinical context warrants; local urgent-cancer referral requirements take precedence.

## Research priorities and framework validation

The framework should first be tested for reproducibility in first-contact settings. A prospective record could capture the presenting problem, the first documented escalation trigger, its risk category, the indicated action, action initiation and completion dates, and any reason for deferral. Independent clinical adjudication should distinguish a missed opportunity from reasonable observation or unavoidable access constraints. The distinction between action initiation and completed access is important when interpreting performance. These proposed measurements have not been validated by the present review.

Evaluation should include patients ultimately found to have benign disease, not only those reaching a sarcoma center. Relevant outcomes include missed malignancy, diagnostic concordance, unplanned procedures, time to completed specialist assessment, symptom burden, function, referral volume, and unnecessary imaging or biopsy. The 2–4-week window requires evaluation rather than assuming benefit. Multicenter implementation studies should assess feasibility, equity, and resource use across different referral systems. Survival analyses should separate interval definitions and account for tumor subtype, grade, anatomical site, symptom severity, and relevant case mix; potential confounders should be distinguished from factors that may mediate an interval effect (9, 10, 12).

## Limitations

This narrative, non-systematic review is intended to support clinical interpretation rather than provide an exhaustive evidence synthesis. Purposive, non-exhaustive literature selection and clinical interpretation introduce selection and citation bias. No formal study-level quality or risk-of-bias assessment was undertaken, and the certainty of the synthesized evidence was not formally graded. Guideline recommendations, observational studies, reviews, and case reports should therefore not be treated as equivalent evidence. The literature is predominantly English-language, although selected Spanish-language and bilingual reports were assessed; non-English-language research and locally published descriptions of diagnostic pathways may be underrepresented.

Much of the cited diagnostic-pathway evidence comes from retrospective or referral-center cohorts. These samples include patients who reached specialist services and may not capture those who were never referred; their case mix and diagnostic resources differ from unselected first-contact practice. Reported symptom frequencies, diagnostic intervals, and procedural outcomes therefore cannot be assumed to apply directly to community or primary care populations. Geographical coverage remains uneven, and differences in referral arrangements, imaging access, pathology expertise, and treatment capacity limit transferability between healthcare systems.

The trigger-to-action framework and its 2–4-week low-risk reassessment interval are author-proposed and have not been prospectively validated; they should not be interpreted as a diagnostic rule or an outcome-proven intervention. The literature also provides no universal diagnostic-interval cutoff or consistent association with survival (10). Evaluation in first-contact settings is needed to measure diagnostic performance, referral burden, missed diagnoses, procedural safety, and patient-reported outcomes before effectiveness or cost-effectiveness can be claimed.

## Conclusion

The contribution of this narrative review is a trigger-to-action framework for decisions before specialist referral, not a replacement for sarcoma guidelines. It links concerning symptom trajectories, imaging uncertainty, and diagnostic discordance to reassessment, further imaging, specialist referral, or coordinated biopsy. The low-risk reassessment window is provisional, and suspected emergencies require immediate assessment. Implementation depends on local diagnostic capacity and reliable handover arrangements. The principal rationale is diagnostic and procedural safety; inconsistent, confounded associations between interval length and survival do not justify a universal deadline or a claim of proven survival benefit. Prospective evaluation should measure both missed diagnoses and the burden of unnecessary escalation.

## References

[1] Franchi A . Epidemiology and classification of bone tumors. Clin cases Miner Bone Metab. (2012) 9:92–5. PMC3476517 23087718

[2] Damron TA Ward WG Stewart A . Osteosarcoma, chondrosarcoma, and Ewing's sarcoma: National Cancer Data Base Report. Clin Orthop Relat Res. (2007) 459:40–7. doi: 10.1097/BLO.0b013e318059b8c9 17414166

[3] Ottaviani G Jaffe N . The epidemiology of osteosarcoma. Cancer Treat Res. (2009) 152:3–13. doi: 10.1007/978-1-4419-0284-9_1 20213383

[4] Strauss SJ Frezza AM Abecassis N Bajpai J Bauer S Biagini R . Bone sarcomas: ESMO-EURACAN-GENTURIS-ERN PaedCan Clinical Practice Guideline for diagnosis, treatment and follow-up. Ann Oncol. (2021) 32:1520–36. doi: 10.1016/j.annonc.2021.08.1995 34500044

[5] Plant J Cannon S . Diagnostic work up and recognition of primary bone tumours: a review. EFORT Open Rev. (2016) 1:247–53. doi: 10.1302/2058-5241.1.000035 28461955 PMC5367572

[6] Widhe B Widhe T . Initial symptoms and clinical features in osteosarcoma and Ewing sarcoma. J Bone Joint Surg Am. (2000) 82:667–74. doi: 10.2106/00004623-200005000-00007 10819277

[7] Liu JF Shanmugavadivel D Ball-Gamble A Walker D . Clinical presentation of bone tumours in children and young people: a systematic review and meta-analysis. Arch Dis Child. (2025) 110:622–9. doi: 10.1136/archdischild-2024-327879 39909718 PMC12320600

[8] Kha ST Sharma J Kenney D Daldrup-Link H Steffner R . Assessment of the interval to diagnosis in pediatric bone sarcoma. Pediatr Emerg Care. (2023) 39:963–7. doi: 10.1097/PEC.0000000000003031 37567167 PMC11805471

[9] Ciechanowicz D Kotrych D Starszak K Prowans P Zacha S Kamiński A . Delay in diagnosis and treatment of bone sarcoma: systematic review. Cancers (Basel). (2025) 17:981. doi: 10.3390/cancers17060981 40149315 PMC11940577

[10] Soomers V Husson O Young R Desar I van der Graaf WTA . The sarcoma diagnostic interval: a systematic review on length, contributing factors and patient outcomes. ESMO Open. (2020) 5:e000592. doi: 10.1136/esmoopen-2019-000592 32079621 PMC7046415

[11] Goedhart LM Gerbers JG Ploegmakers JJW Jutte PC . Delay in diagnosis and its effect on clinical outcome in high-grade sarcoma of bone: a referral oncological centre study. Orthop Surg. (2016) 8:122–8. doi: 10.1111/os.12239 27384720 PMC6584288

[12] Weller D Vedsted P Rubin G Walter FM Emery J Scott S . The Aarhus statement: improving design and reporting of studies on early cancer diagnosis. Br J Cancer. (2012) 106:1262–7. doi: 10.1038/bjc.2012.68 22415239 PMC3314787

[13] Goyal S Roscoe J Ryder WDJ Gattamaneni HR Eden TOB . Symptom interval in young people with bone cancer. Eur J Cancer. (2004) 40:2280–6. doi: 10.1016/j.ejca.2004.05.017 15454254

[14] Gerrand C Amary F Anwar HA Brennan B Dileo P Kalkat MS . UK guidelines for the management of bone sarcomas. Br J Cancer. (2025) 132:32–48. doi: 10.1038/s41416-024-02868-4 39550489 PMC11723950

[15] Blay JY Palmerini E Bollard J Aguiar S Angel M Araya B . SELNET clinical practice guidelines for bone sarcoma. Crit Rev Oncol Hematol. (2022) 174:103685. doi: 10.1016/j.critrevonc.2022.103685 35460913

[16] Biermann JS Hirbe A Ahlawat S Bernthal NM Binitie O Boles S . Bone cancer, version 2.2025, NCCN clinical practice guidelines in oncology. J Natl Compr Canc Netw. (2025) 23:e250017. doi: 10.6004/jnccn.2025.0017 40203873

[17] Dyrop HB Vedsted P Raedkjaer M Safwat A Keller J . Imaging investigations before referral to a sarcoma center delay the final diagnosis of musculoskeletal sarcoma. Acta Orthop. (2017) 88:211–6. doi: 10.1080/17453674.2016.1278113 28077058 PMC5385118

[18] Ashwood N Witt JD Hallam PJ Cobb JP . Analysis of the referral pattern to a supraregional bone and soft tissue tumour service. Ann R Coll Surg Engl. (2003) 85:272–6. doi: 10.1308/003588403766275015 12855033 PMC1964394

[19] Siddiqui YS Sherwani MKA Khan AQ Zahid M Abbas M Asif N . Neglected orthopedic oncology: causes, epidemiology and challenges for management in developing countries. Indian J Cancer. (2015) 52:325–9. doi: 10.4103/0019-509X.176737 26905128

[20] Bajpai J Khanna N Vora T Gulia A Laskar S Puri A . Analysis of bone and soft-tissue sarcomas registered during the year 2012 at Tata Memorial Hospital, Mumbai, with clinical outcomes. Indian J Cancer. (2018) 55:37–44. doi: 10.4103/ijc.IJC_481_17 30147091

[21] Lisenda L Linda ZA Snyman FPJ Kyte RD Lukhele M . Osteosarcoma patient outcomes at a South African tertiary hospital. S Afr Med J. (2017) 107:754–7. doi: 10.7196/SAMJ.2017.v107i9.11424 28875882

[22] Vasquez L Silva J Chavez S Zapata A Diaz R Tarrillo F . Prognostic impact of diagnostic and treatment delays in children with osteosarcoma. Pediatr Blood Cancer. (2020) 67:e28180. doi: 10.1002/pbc.28180 31925940

[23] Pan KL Chan WH Chia YY . Initial symptoms and delayed diagnosis of osteosarcoma around the knee joint. J Orthop Surg (Hong Kong). (2010) 18:55–7. doi: 10.1177/230949901001800112 20427835

[24] Abdelmabood S Kandil S Megahed A Fouda A . Delays in diagnosis and treatment among children with cancer: Egyptian perspective. East Mediterr Health J. (2017) 23:422–9. doi: 10.26719/2017.23.6.422 28836655

[25] Brand NR Qu LG Chao A Ilbawi AM . Delays and barriers to cancer care in low- and middle-income countries: a systematic review. Oncologist. (2019) 24:e1371–80. doi: 10.1634/theoncologist.2019-0057 31387949 PMC6975966

[26] Fossum CC Breen WG Sun PY Retzlaff AA Okuno SH . Assessment of familiarity with work-up guidelines for bone and soft tissue sarcoma among primary care practitioners in Minnesota. Mayo Clin Proc Innov Qual Outcomes. (2020) 4:143–9. doi: 10.1016/j.mayocpiqo.2019.12.002 32280924 PMC7140016

[27] Shanmugavadivel D Liu JF Ball-Gamble A Polanco A Vedhara K Nathan P . Childhood bone tumours in primary care: helping GPs to identify 'the needle in the haystack'. Br J Gen Pract. (2023) 73:377–9. doi: 10.3399/bjgp23X734673 37500459 PMC10405944

[28] George A Grimer R . Early symptoms of bone and soft tissue sarcomas: could they be diagnosed earlier? Ann R Coll Surg Engl. (2012) 94:261–6. doi: 10.1308/003588412X13171221590016 22613305 PMC3957506

[29] Durbin M Randall RL James M Sudilovsky D Zoger S . Ewing's sarcoma masquerading as osteomyelitis. Clin Orthop Relat Res. (1998), 176–85. doi: 10.1097/00003086-199812000-00023 9917715

[30] Gazendam A Popovic S Parasu N Ghert M . Chondrosarcoma: a clinical review. J Clin Med. (2023) 12:2506. doi: 10.3390/jcm12072506 37048590 PMC10095313

[31] Douis H Parry M Vaiyapuri S Davies AM . What are the differentiating clinical and MRI-features of enchondromas from low-grade chondrosarcomas? Eur Radiol. (2018) 28:398–409. doi: 10.1007/s00330-017-4947-0 28695356

[32] Wurtz LD Peabody TD Simon MA . Delay in the diagnosis and treatment of primary bone sarcoma of the pelvis. J Bone Joint Surg Am. (1999) 81:317–25. doi: 10.2106/00004623-199903000-00003 10199269

[33] van der Waal I . Professional diagnostic delay in osteosarcomas of the jaws. Med Oral Patol Oral Cir Bucal. (2020) 25:e834–7. doi: 10.4317/medoral.24334 33125360 PMC7648920

[34] Ahlawat S Lenchik L Baker JC Allen H Banks J Expert Panel on Musculoskeletal Imaging . ACR appropriateness criteria suspected primary bone tumors: 2024 update. J Am Coll Radiol. (2025) 22:S440–54. doi: 10.1016/j.jacr.2025.02.020 40409893

[35] Hoffman RJ Stanborough RO Garner HW . Diagnostic imaging approach to solitary bone lesions. Semin Roentgenol. (2022) 57:241–51. doi: 10.1053/j.ro.2022.01.005 35842245

[36] Chang CY Garner HW Ahlawat S Amini B Bucknor MD Flug JA . Society of Skeletal Radiology-white paper. Guidelines for the diagnostic management of incidental solitary bone lesions on CT and MRI in adults: bone reporting and data system (Bone-RADS). Skeletal Radiol. (2022) 51:1743–64. doi: 10.1007/s00256-022-04022-8 35344076 PMC9283187

[37] Ferguson JL Turner SP . Bone cancer: diagnosis and treatment principles. Am Fam Physician. (2018) 98:205–13. 30215968

[38] Mankin HJ Mankin CJ Simon MA . The hazards of the biopsy, revisited. J Bone Joint Surg Am. (1996) 78:656–63. doi: 10.2106/00004623-199605000-00004 8642021

[39] Traina F Errani C Toscano A Pungetti C Fabbri D Mazzotti A . Current concepts in the biopsy of musculoskeletal tumors: AAOS exhibit selection. J Bone Joint Surg Am. (2015) 97:e7. doi: 10.2106/JBJS.N.00661 25609446

[40] Oliveira MP Lima PMA Silva HJ Mello RJV . Neoplasm seeding in biopsy tract of the musculoskeletal system: a systematic review. Acta Ortop Bras. (2014) 22:106–10. doi: 10.1590/1413-78522014220200422 24899866 PMC4031257

[41] Garcia JG Marques DS Viola DCM Petrilli MT Alves MTS Jesus-Garcia Filho R . Biopsy path contamination in primary bone sarcomas. Rev Bras Ortop (Sao Paulo). (2019) 54:33–6. doi: 10.1016/j.rbo.2017.09.006 31363240 PMC6424802

[42] Ferreira FBMD Puchnick A Garcia DL Regacini R Perez P Rosa Pinto MB . Image-guided percutaneous needle biopsy for Benign and Malignant bone tumors: systematic review and meta-analysis. J Vasc Interv Radiol. (2023) 34:623–32.e2. doi: 10.1016/j.jvir.2022.12.467 36587804

[43] Crenn V Vezole L Bouhamama A Meurgey A Karanian M Marec-Bérard P . Percutaneous core needle biopsy can efficiently and safely diagnose most primary bone tumors. Diagnostics (Basel). (2021) 11:1552. doi: 10.3390/diagnostics11091552 34573895 PMC8469906

[44] Crenn V Lataste F Galmiche L Le Nail LR Ropars M Blay JY . Enhanced accuracy and reduced delay in diagnosing bone tumors within an expert sarcoma network: A nationwide study. Eur J Surg Oncol. (2024) 50:108271. doi: 10.1016/j.ejso.2024.108271 38522331

[45] Gao ZH Yin JQ Liu DW Meng QF Li JP . Preoperative easily misdiagnosed telangiectatic osteosarcoma: clinical-radiologic-pathologic correlations. Cancer Imaging. (2013) 13:520–6. doi: 10.1102/1470-7330.2013.0042 24334494 PMC3864225

[46] Nakamura T Sugaya J Naka N Kobayashi H Okuma T Kunisada T . Standard treatment remains the recommended approach for patients with bone sarcoma who underwent unplanned surgery: report from the Japanese Musculoskeletal Oncology Group. Cancer Manag Res. (2020) 12:10017–22. doi: 10.2147/CMAR.S270178 33116858 PMC7567545

[47] Kim MS Lee SY Cho WH Song WS Koh JS Lee JA . Prognostic effects of doctor-associated diagnostic delays in osteosarcoma. Arch Orthop Trauma Surg. (2009) 129:1421–7. doi: 10.1007/s00402-009-0851-7 19280203

[48] Blay JY Soibinet P Penel N Bompas E Duffaud F Stoeckle E . Improved survival using specialized multidisciplinary board in sarcoma patients. Ann Oncol. (2017) 28:2852–9. doi: 10.1093/annonc/mdx484 29117335 PMC5834019

[49] Blay JY Honoré C Stoeckle E Meeus P Jafari M Gouin F . Surgery in reference centers improves survival of sarcoma patients: a nationwide study. Ann Oncol. (2019) 30:1143–53. doi: 10.1093/annonc/mdz124 31081028 PMC6637376

[50] Brasme JF Chalumeau M Oberlin O Valteau-Couanet D Gaspar N . Time to diagnosis of Ewing tumors in children and adolescents is not associated with metastasis or survival: a prospective multicenter study of 436 patients. J Clin Oncol. (2014) 32:1935–40. doi: 10.1200/JCO.2013.53.8058 24841977

[51] Sasi A Ganguly S Biswas B Pushpam D Kumar A Agarwala S . Determinants and impact of diagnostic interval in bone sarcomas: A retrospective cohort study. Pediatr Blood Cancer. (2023) 70:e30135. doi: 10.1002/pbc.30135 36524611

[52] Hu J Zhang C Zhu K Zhang L Cai T Zhan T . Is increased symptom interval associated with advanced stage and poorer outcome? A prospective multicenter study of 220 patients with osteosarcoma around the knee. Cancer Epidemiol. (2020) 67:101776. doi: 10.1016/j.canep.2020.101776 32645592

[53] Yoshida S Celaire J Pace C Taylor C Kaneuchi Y Evans S . Delay in diagnosis of primary osteosarcoma of bone in children: Have we improved in the last 15 years and what is the impact of delay on diagnosis? J Bone Oncol. (2021) 28:100359. doi: 10.1016/j.jbo.2021.100359 33898215 PMC8056435

[54] Clara-Altamirano MA García-Ortega DY Álvarez-Cano A Velázquez-Rodríguez S Lizcano-Suárez AR Rosas LC . Delayed diagnosis of osteosarcoma in adults: a prognostic factor to be considered. Acta Ortop Mex. (2024) 38:164–71. doi: 10.35366/115811 38862146

[55] Sun L Li Y Zhang J Li H Li B Ye Z . Prognostic value of pathologic fracture in patients with high grade localized osteosarcoma: a systemic review and meta-analysis of cohort studies. J Orthop Res. (2015) 33:131–9. doi: 10.1002/jor.22734 25231396

[56] National Cancer Grid . Bone and Soft Tissue Tumors Management Guidelines 2019 (2019). Mumbai: National Cancer Grid. Available online at: https://www.ncgIndia.org/assets/ncg-guidelines-2019/ncg-guidelines-for-bst-2019.pdf (Accessed September 3, 2026).

[57] Mittal A Pushpam D Ganguly S Kumar VS Khan SA Bakhshi S . Controversies and challenges in the management of osteosarcoma-an Indian perspective. Indian J Surg Oncol. (2022) 13:939–55. doi: 10.1007/s13193-021-01486-3 36687236 PMC9845467

[58] Li H Zheng S Yu W Huang W Yao Y Shen Z . Symptom interval of osteosarcoma around the knee joint: an analysis of 82 patients of a single institute. Eur J Cancer Care (Engl). (2016) 25:849–54. doi: 10.1111/ecc.12406 26568464

[59] Kotrych D Ciechanowicz D Pawlik J Brodecki A Białomyzy A Prowans P . Delay in diagnosis and treatment of primary bone tumors. Ortop Traumatol Rehabil. (2023) 25:9–22. doi: 10.5604/01.3001.0053.4026 38078348

[60] Gómez-Martínez R Verdugo-Robles MÁ López-Márquez G Santiago-Aguilar AI Romo-Rubio H . Osteosarcoma in pre-adolescents: diagnostic interval, metastasis frequency, and treatment abandonment. Bol Med Hosp Infant Mex. (2025) 82:167–71. doi: 10.24875/BMHIM.23000147 40706085

